# Identification of blood biomarkers in glioblastoma by SWATH mass spectrometry and quantitative targeted absolute proteomics

**DOI:** 10.1371/journal.pone.0193799

**Published:** 2018-03-07

**Authors:** Eisuke Miyauchi, Takuya Furuta, Sumio Ohtsuki, Masanori Tachikawa, Yasuo Uchida, Hemragul Sabit, Wataru Obuchi, Tomoko Baba, Michitoshi Watanabe, Tetsuya Terasaki, Mitsutoshi Nakada

**Affiliations:** 1 Division of Membrane Transport and Drug Targeting, Graduate School of Pharmaceutical Sciences, Tohoku University, Sendai, Miyagi, Japan; 2 Department of Pathology, Kurume University School of Medicine, Kurume, Fukuoka, Japan; 3 Department of Neurosurgery, Graduate School of Medical Science, Kanazawa University, Kanazawa, Ishikawa, Japan; 4 Department of Pharmaceutical Microbiology, Faculty of Life Sciences, Kumamoto University, Kumamoto, Kumamoto, Japan; Northwestern University, UNITED STATES

## Abstract

Molecular biomarkers in blood are needed to aid the early diagnosis and clinical assessment of glioblastoma (GBM). Here, in order to identify biomarker candidates in plasma of GBM patients, we performed quantitative comparisons of the plasma proteomes of GBM patients (n = 14) and healthy controls (n = 15) using SWATH mass spectrometry analysis. The results were validated by means of quantitative targeted absolute proteomics analysis. As a result, we identified eight biomarker candidates for GBM (leucine-rich alpha-2-glycoprotein (LRG1), complement component C9 (C9), C-reactive protein (CRP), alpha-1-antichymotrypsin (SERPINA3), apolipoprotein B-100 (APOB), gelsolin (GSN), Ig alpha-1 chain C region (IGHA1), and apolipoprotein A-IV (APOA4)). Among them, LRG1, C9, CRP, GSN, IGHA1, and APOA4 gave values of the area under the receiver operating characteristics curve of greater than 0.80. To investigate the relationships between the biomarker candidates and GBM biology, we examined correlations between plasma concentrations of biomarker candidates and clinical presentation (tumor size, progression-free survival time, or overall survival time) in GBM patients. The plasma concentrations of LRG1, CRP, and C9 showed significant positive correlations with tumor size (R^2^ = 0.534, 0.495, and 0.452, respectively).

## Introduction

Glioblastoma (GBM) is the most malignant (WHO grade IV) and most common tumor of the brain. Nearly 90% of GBM develops rapidly de novo without isocitrate dehydrogenase (IDH) mutations, with no clinical or histologic evidence of a less malignant precursor lesion. Even with improved treatment modalities, the median survival of these patients is only about 15 months [1,2].

Non-invasive imaging techniques are usually performed for GBM diagnosis, and implementation of advanced imaging modalities has improved the neuroradiological diagnostic accuracy. Still, complete specificity for differentiation of brain tumors and detection of minor differences in tumor size and behavior are difficult using such imaging approaches. Diagnosis using tumor tissue extracted by neurosurgical intervention (biopsy or resection) may not always be feasible. Reliable blood biomarkers could be helpful for the management of GBM patients, e.g., by enabling early diagnosis, by facilitating neuroradiological differential diagnosis at initial presentation, by aiding the planning of surgical interventions, and by allowing monitoring of the disease course. However, no blood biomarkers have been established for routine clinical management of GBM patients, although some previous studies have identified blood biochemical alterations with potential clinical utility in GBM [3,4]. Strojnik *et al*. defined C-reactive protein (CRP) as a serological prognostic marker for GBM [5]. Other individual serum biomarkers include chitinase-3-like protein 1 [6,7], glial fibrillary acidic protein [8], matrix metalloproteinase-9 [6], epidermal growth factor receptor [9], CD14 [10], a proliferation-inducing ligand [11], and insulin-like growth factor binding protein-2 [12].

Mass spectrometry (MS)-based proteomic analysis of human clinical blood is a powerful tool to investigate cancer biomarkers [13]. Numerous clinical studies of GBM have been reported over the past decade using various quantitative approaches [14–18]. SWATH-MS is an emerging quantitative technique that combines a highly specific data-independent acquisition (DIA) method with a novel targeted data extraction strategy to mine the resulting fragment ion data sets [19]. SWATH-MS analysis offers several advantages, including high reproducibility and reliability of quantitative information, in discovery proteomics [20,21]. However, it has not yet been applied to study GBM proteomics, to our knowledge.

One of the greatest advantages of SWATH-MS is that it is a label-free analysis, which can be employed with conventional and comprehensive liquid chromatography-tandem mass spectrometry (LC-MS/MS), but nevertheless, the possibility of false-positive results caused by sample-dependent ion-suppression cannot be excluded. Hence, accurate and reliable absolute quantification is essential to validate biomarker candidates identified by SWATH-MS screening. We have established a protein quantification method using LC-MS/MS, called quantitative targeted absolute proteomics (QTAP), which enables us to simultaneously determine absolute protein expression levels [22,23].

Thus, the purpose of this study was to identify plasma biomarker candidates for GBM patients by using a combination of SWATH-MS and QTAP. With this approach, in order to estimate the origin of up-regulated biomarker candidates in GBM plasma, we examined whether or not the up-regulated biomarker candidates are also elevated in GBM tissues, and also whether or not they are detectable in cyst fluid. Moreover, to investigate the relationships between biomarker candidates and GBM biology, we examined the correlations between the concentrations of biomarker candidates in plasma and the clinical presentation (tumor size, progression-free survival time (PFS), or overall survival time (OS)) of GBM patients. In addition, PFS and OS probabilities in GBM patients with low or high biomarker candidate plasma levels were examined.

## Materials and methods

### Ethics statement

This study was done with written informed consent from every subject. The research protocols for the present study were reviewed and approved by the ethics committees of the Graduate School of Medical Sciences, Kanazawa University (Number 1363) and the Graduate School of Pharmaceutical Sciences, Tohoku University (Number 13–04).

### Clinical samples

As shown in Table 1, plasma and tumor tissue samples were obtained from 14 GBM patients. Cyst fluid samples were obtained from three of the 14 GBM patients (P1, P4, and P10). Plasma samples were taken before treatment. Fresh tumor tissue samples were taken from GBM patients undergoing therapeutic removal of brain tumors under an institutional review board-approved protocol. Histological diagnosis was made by standard light-microscopic evaluation of sections stained with hematoxylin and eosin. The diagnosis of all patients are GBM, IDH-wildtype according to the revised WHO criteria for tumors of the central nervous system [24]. For comparative proteomic analysis, plasma samples were also obtained from 15 healthy subjects. Two noncancerous brain tissue samples (ABS352JK00909 (B1) and ABS2467970101 (B2)) were purchased from Analytical Biological Services Inc. (Wilmington, Delaware). All samples were stored at −80°C. For the tumor volumetric analysis, contrast-enhanced T1-weighted images were used. Our volumetric method was previously described in detail [25].

**Table 1:** Characteristics of subjects.

Subject ID	Age (years)	Sex	Tumor Size (cm^3^)	PFS (months)	OS (months)
**Glioblastoma (WHO grade IV)**					
P1*	61	M	13.6	6.7	16.4
P2	55	M	4.5	13.9	35.4
P3	58	M	8.5	0.6	0.6
P4*	43	M	90.3	No recurrence	Survival (44.2)
P5	79	F	30.2	8.5	11.3
P6	83	M	81.5	8.0	11.0
P7	68	F	25.3	6.8	22.9
P8	71	M	10.5	9.6	21.0
P9	64	M	21.2	No recurrence	Survival (38.2)
P10*	70	M	50.2	3.5	8.0
P11	80	F	31.5	16.8	21.9
P12	54	M	55.1	No recurrence	Survival (36.5)
P13	61	M	20.7	8.4	15.0
P14	60	M	13.6	10.2	24.6
**Healthy plasma**					
C1	44	M			
C2	42	M			
C3	40	M			
C4	35	M			
C5	32	M			
C6	59	F			
C7	54	F			
C8	34	F			
C9	29	M			
C10	27	M			
C11	30	M			
C12	63	F			
C13	54	F			
C14	34	M			
C15	31	M			
**Noncancerous brain tissue**					
B1	56	F			
B2	70	M			

All glioblastoma (GBM) patients had isocitrate dehydrogenase-wildtype. Plasma and tumor tissue samples were obtained from all GBM patients. Cyst fluid samples were obtained from three GBM patients shown in bold with an asterisk (*). The above patient information is as of August 1, 2015. There was a statistically significant difference (p = 4.85×10^−6^) in age between GBM patients and healthy controls. There was no statistically significant difference in gender between GBM patients and healthy controls. F, female; M, male; OS, overall survival time; PFS, progression-free survival time

### Preparation of whole tissue lysate, cytosol, microsome, and plasma membrane fractions

Whole tissue lysate, cytosol, microsome, and plasma membrane fractions were prepared as described previously [26], with several modifications as follows. Frozen tissues were homogenized using a Potter-Elvehjem homogenizer in buffer A containing (in mM) 10 Tris-HCl (pH 7.4), 10 NaCl, 1.5 MgCl_2_, 1 phenylmethylsulfonyl fluoride, and a protease inhibitor cocktail (Sigma Chemical Co., Saint Louis, Missouri). The homogenates were additionally subjected to nitrogen cavitation (450 psi for 15 min at 4°C) in buffer A, and a part of the solution was stored at −80°C as whole tissue lysate. The remaining homogenates were centrifuged at 10,000g for 10 min at 4°C twice, and the supernatants were ultracentrifuged at 100,000g for 40 min at 4°C. The supernatants were stored at −80°C as cytosol fractions.

### Sample preparation for LC-MS/MS analysis

Plasma, cyst fluid, whole cell lysate, cytosol, and microsome fractions were solubilized in 8 M urea in 100 mM Tris-HCl (pH 8.5), and S-carbamoylmethylated with dithiothreitol and iodoacetamide as described [27]. The S-carbamoylmethylated samples were diluted five-fold with protease MAX surfactant (Promega, Madison, Wisconsin; final concentration 0.05%) and 100 mM Tris-HCl (pH 8.5), and treated with lysyl endopeptidase (Wako Pure Chemical Industries, Osaka, Japan) at an enzyme/substrate ratio of 1:100 (plasma and cyst fluid), 1:20 (whole cell lysate and microsome fractions), or 1:10 (cytosol fractions) at 30°C for 3 h. Subsequently, samples were digested with sequence-grade modified trypsin (Promega) at an enzyme/substrate ratio of 1:100 (plasma and cyst fluid), 1:20 (whole cell lysate and microsome fractions), or 1:10 (cytosol fractions) at 37°C for 16 h.

Plasma membrane fractions were solubilized in denaturing buffer (7 M guanidine hydrochloride, 500 mM Tris–HCl (pH 8.5), 10 mM EDTA), and the proteins were S-carbamoylmethylated with dithiothreitol and iodoacetamide as described [22]. The alkylated proteins were precipitated with a mixture of methanol, chloroform and water. The precipitates were dissolved in 6 M urea in 100 mM Tris–HCl (pH 8.5). The dissolved samples were diluted five-fold with protease MAX surfactant (Promega; final concentration 0.05%) and 100 mM Tris-HCl (pH 8.5), and treated with lysyl endopeptidase (Wako Pure Chemical Industries) at an enzyme/substrate ratio of 1:100 at 30°C for 3 h. Subsequently, samples were digested with sequence-grade modified trypsin (Promega) at an enzyme/ substrate ratio of 1:100 at 37°C for 16 h.

The tryptic digests were desalted with SDB-Tip and GC-Tip (GL Science, Tokyo, Japan). For library construction, isoelectric point-based peptide separation was performed. Some desalted samples (plasma obtained from subject P14 and cyst fluid obtained from subject P1) were divided into 12 fractions with a 3–10 linear pH range by a 3100 OFFGEL Fractionator (Agilent Technologies, Böblingen, Baden-Württemberg, Germany). The fractionated samples were desalted with SDB-Tip and GC-Tip (GL Science).

### Conditions of data-dependent acquisition (DDA) and SWATH-MS analyses

For library construction, the desalted peptides were injected into a nano-LC ultra 2D plus (Eksigent Technologies, Dublin, California) coupled with an electrospray-ionization Triple TOF 5600 mass spectrometer (SCIEX, Framingham, Massachusetts). Using a cHiPLC nanoflex system (Eksigent Technologies) with the nano-LC, injected peptides (5 μL; 0.02 μL plasma or 0.2 μg protein/μL) were loaded onto a trap column (200 μm x 6 mm, ReproSil-Pur 3 μm, C18-AQ 120A°) (Eksigent Technologies) and separated on an analytical column (75 μm x 15 cm, ReproSil-Pur 3 μm, C18-AQ 120A°) (Eksigent Technologies). The flow rates were 2 μL/min (six minutes run-time) for loading on the trap column and 300 nL/min for separation on the analytical column. The injected peptides were eluted in (A = 0.1% formic acid in Milli-Q water, B = 0.1% formic acid in 100% acetonitrile) 0–20%B (0–60 min), changed to 20–40%B (60–75 min), increased to 40–100%B (75–77 min), maintained at 100%B (77–82 min), reduced to 0%B (82–84 min), and then maintained at 0%B (84–115 min). For data acquisition, all MS spectra (full scan type) with a mass range (m/z) of 300–1008 were recorded using DDA in the positive ionization mode. For fragmentation, a maximum of 20 intense precursor ions per cycle with a charge state between 2+ to 5+ were selected. The collision energy spread was set at 5 eV. Consequently, product ions or MS/MS fragments in the range of 100–1600 m/z were collected. To achieve optimum efficiency of fragmentation, DDA was used to automatically control the collision energy (CE) based on Eq 1.

CE=0.044×(precursorionm/z)+4 (Eq 1)

In SWATH acquisition, the parameters were essentially the same as those described by Gillet *et al*. [19]. With the same chromatographic conditions used in the DDA run described above and 59 fixed 13 Da wide windows (including 1 Da for window overlap), the precursor mass range of 300–1008 Da was covered. The CE for each window was determined from Eq 1 based on the appropriate CE for a 2+ ion centered in the window with a spread of 5 eV. The high-sensitivity mode was used, allowing accurate extraction of the fragment ion masses.

### Construction of spectral library for the SWATH-MS analysis for biomarker protein discovery in GBM patients’ plasma

We chose SWATH-MS analysis for the present purpose because of its high reproducibility and quantitative reliability in discovery proteomics studies [20,21]. Although the peaks generated by peptide fragments were simultaneously detected by Triple TOF 5600 mass spectrometry using the DIA mode in the SWATH-MS analysis, each peak needs to be assigned based on a spectral library consisting of precursor ion masses, fragment ion masses, fragment ion intensities, and retention times [19]. Therefore, it is essential that the constructed spectral library includes information about all biomarker protein candidates. We assumed that biomarker proteins in the plasma of GBM patients are produced in the GBM tissues and released into the cyst fluid and the plasma. Because SWATH-MS analysis is more sensitive in identifying and quantifying peptides compared with DDA used for library construction in side-by-side analyses on the same instrument [19], it might be worth fractionating the samples used to generate the library before the analysis. A decrease in the amount of co-existing peptides during the ionization process will be effective for increasing the number of identified proteins (by decreasing ion-suppression effects). Therefore, we separated plasma and cyst fluid of GBM patients by means of preparative isoelectric focusing (IEF) into 12 different fractions with a 3–10 linear pH range. Subcellular fractionation enables the in-depth analysis of biomolecules by reducing the complexity of the protein mixture. The GBM tissue homogenate was separated into plasma membrane, microsome and cytosol fractions. Soluble proteins in cytosol fractions and membrane proteins in plasma membrane and microsome fractions are potentially secreted, leaked, and shed from cells into the circulation, while some are actively secreted as microvesicles, such as exosomes. DDA data were analyzed using the Paragon algorithm of ProteinPilot Version 4.5 (SCIEX), and the UniProt Human proteome database (release2016_02, entries) was searched. The six user-defined options included (i) cysteine alkylation, iodoacetamide; (ii) digestion, trypsin digestion; (iii) special factors, none; (iv) species, Homo sapiens; (v) identification focus, biological modification; and (vi) search effort, thorough identification search. The protein identification confidence for the dataset was evaluated versus the false discovery rate (FDR) obtained in a concomitant search of the UniProt Human proteome database for the reverse sequences. The FDR values were all lower than 1%. Table 2 summarizes the number of proteins and peptides identified in each sample analysis. Excluding overlapped peptides or proteins, 20,066 peptides and 2,930 proteins were included in the spectral library (Table 2). In IEF-fractionated plasma of a GBM patient, 78 novel proteins were identified in addition to those identified in plasma of GBM patients by DDA. In IEF-fractionated cyst fluid of a GBM patient, 553 proteins were newly identified in addition to the proteins identified in cyst fluid of GBM patients by DDA. In the cytosol, microsome and plasma membrane fractions of GBM tissue, 169, 610 and 1,889 proteins were newly identified in addition to the proteins identified in whole tissue lysates of GBM tissue by DDA, respectively, of which 27, 39 and 1,237 proteins were unique, respectively (S1 Fig). The SWATH-MS data was also analyzed with an in-house spectral library generated from DDA data acquired in other experiments (http://www.peptideatlas.org/, Identifier: PASS01107).

**Table 2:** Total number of proteins and peptides in spectral library.

GBM patients	Number of specimens	Number of identified	
		Proteins	Peptides
Plasma	14	216	1,689
Plasma (IEF)	1	282	2,642
Cyst fluid	3	266	1,074
Cyst fluid (IEF)	1	811	4,331
**Tumor tissue**			
Whole tissue lysate	14	761	2,236
Cytosol fraction	14	684	2,521
Microsome fraction	14	1,280	4,632
Plasma membrane fraction	13	2,590	14,523
**Total number in spectral library**		**2,930**	**20,066**

Plasma obtained from subject P14 and cyst fluid obtained from subject P1 were divided into 12 fractions with a 3–10 linear pH range by preparative isoelectric focusing (IEF). Only 13 plasma membrane fractions of glioblastoma (GBM) patients could be obtained, because the tissue volume of subject P13 was too small for preparing plasma membrane fraction. Modified peptides were not included in the number of identified peptides. Only proteins registered in SWISS-PROT were included in the number of identified proteins. All isoforms of a protein were counted as one.

### SWATH-MS data analysis

Spectral alignment and targeted data extraction of DIA samples were performed with the SWATH Processing Micro App in Peakview (Version 2.0, SCIEX) using the spectral library generated as described above. DIA raw files were loaded in unison using an extraction window of 6 min and the following parameters: 999 peptides, 99 transitions and peptide confidence of > 99%, FDR of < 1% with at least one sample at the peptide level, excluding shared peptides and XIC width set at 50 ppm. After data processing, raw data including peak area and retention time were exported from Peak View to Excel.

### Synthesis of standard and internal standard peptides of biomarker candidates for GBM

cDNAs encoding an N-terminal Strep-tag, a C-terminal HAT-tag, N- and C-terminal reference peptides, and tandemly linked peptides of biomarker candidates for GBM were synthesized and subcloned into pET-17b. Recombinant proteins were expressed using a cell-free expression system (Taiyo Nippon Sanso Corporation, Tokyo, Japan). Plasmid and working solution were mixed and incubated for 16 h at 30°C. For stable isotope labeling, amino acid mixture containing Lys, Arg-^13^C,^15^N was incubated with the expression system. After the reaction, the solution was centrifuged at 4°C and 17,800 × g for 5 min. The pellet were collected and suspended in PBS buffer, then solubilized in denaturing buffer (7 M guanidine hydrochloride, 100 mM Tris–HCl (pH 8.0)). The solution was centrifuged at 4°C and 17,800 × g for 10 min. The supernatant was diluted 10-fold with wash buffer A (150 mM Tris–HCl, 100 mM NaCl (pH 8.0)). The diluted solution was centrifuged at 4°C and 10,000 × g for 10 min. The supernatant was loaded onto a Strep-Tactin Sepharose column (IBA Lifesciences, Göttingen, Niedersachsen, Germany), which was washed with two column volumes of wash buffer A three times. The recombinant proteins were eluted with 0.5 column volumes of elution buffer B (7 M guanidine hydrochloride, 150 mM Tris–HCl, 100 mM NaCl (pH 8.0)) six times. The eluted solutions including recombinant proteins were checked by SDS-PAGE, loaded onto a HisPur cobalt spin column (Thermo Fisher Scientific, Waltham, Massachusetts), and then inverted in a mixer at 4°C for 30 min. The column was centrifuged at 4°C and 700 × g for 2 min, then washed with two column volumes of wash buffer C (6 M guanidine hydrochloride, 10 mM imidazole, 50 mM Tris–HCl, 300 mM NaCl (pH 8.0)) by centrifugation at 4°C and 700 × g for 2 min three times. The recombinant proteins were eluted with one column volume of elution buffer D (6 M guanidine hydrochloride, 150 mM imidazole, 50 mM Tris–HCl, 300 mM NaCl (pH 8.0)) by centrifugation at 4°C and 700 × g for 2 min five times. The eluted solutions including recombinant proteins were checked by SDS-PAGE, dialyzed against 50 mM ammonium bicarbonate in dialysis tubes (MWCO = 10,000) overnight at 4°C with three outside buffer changes, and then lyophilized to a powder. The lyophilized samples were solubilized in 48 mM sodium lauroyl sarcosinate. Protein concentrations were determined by the Lowry method using the DC protein assay reagent (Bio-Rad Laboratories, Hercules, California). The solubilized samples were digested with lysyl endopeptidase and trypsin according to a phase-transfer surfactant protocol [28]. In order to estimate the concentrations of peptides derived from recombinant proteins using the N- and C-terminal reference peptides, the tryptic digests were mixed with internal standard peptides for the reference peptides, and desalted with SDB-Tip and GC-Tip (GL Science). The concentrations of peptides derived from recombinant proteins were calculated as the mean of the quantitative values obtained from N- and C-terminal reference peptides by QTAP analysis.

### LC-MS/MS-based QTAP analysis

Simultaneous protein quantitation of target molecules was performed by using the nanoLC-Triple TOF 5600 with parallel reaction monitoring (PRM) as described previously [26], with several modifications as follows. Using a cHiPLC nanoflex system (Eksigent Technologies) with the nano-LC, peptides (5 μL; 0.02 μL plasma or 0.2 μg protein/μL) were loaded onto a trap column (200 μm x 6 mm, ReproSil-Pur 3 μm, C18-AQ 120A°) (Eksigent Technologies) and separated on an analytical column (75 μm x 15 cm, ReproSil-Pur 3 μm, C18-AQ 120A°) (Eksigent Technologies). The flow rates were 2 μL/min (six minutes run-time) for loading on the trap column and 300 nL/min for separation on the analytical column. The peptides were eluted in (A = 0.1% formic acid in Milli-Q water, B = 0.1% formic acid in 100% acetonitrile) 0–40%B (0–40 min), increased to 40–100%B (40–41 min), maintained at 100%B (41–50 min), reduced to 0%B (50–50.1 min), and then maintained at 0%B (50.1–80 min). The ion counts in the chromatograms were determined by using an auto analysis system established our laboratory [27,29]. Peak identification was based on the fact that the unlabeled peptides showed identical retention times to the corresponding labeled peptides, and the peak area counts were greater than 1,000 for LC-MS/MS with nanoLC analysis.

### Statistical analysis

The F-test was performed to assess the equality of variance between two groups. Student’s t-test was used to determine the statistical significance of differences between two groups. Both tests were performed by Excel software. Cohen’s d effect size was calculated from mean quantitative values (M), standard deviation of quantitative values (SD), and sample size (N) of the case group or control group (ctl) based on the following formula:

$$d=\frac{\left|M_{case}-M_{ctl}\right|}{\sqrt{\frac{(N_{case}-1){SD}_{case}^{2}+(N_{ctl}-1){SD}_{ctl}^{2}}{N_{case}+N_{ctl}-2}}}$$

Fisher’s exact test was used to identify statistically significant differences in gender between two groups. Receiver operating characteristics (ROC) curves were created by plotting sensitivity (y-axis) and 1−specificity (x-axis) at various thresholds. The optimal thresholds for biomarker candidates were determined as the points with minimum distance from 100% sensitivity and 100% specificity in the ROC curves. ROC analysis was performed for GBM plasma against healthy plasma. Pearson correlational analysis was run on comparisons between plasma protein concentration and total protein amount in the tumor tissue, tumor size, PFS, or OS among the GBM patients. PFS and OS probabilities were calculated by Kaplan–Meier analysis. The subgroups in Kaplan–Meier analysis were compared using the log-rank test. ROC analysis, Pearson correlational analysis, Kaplan–Meier analysis and Fisher’s exact test were performed using R project (http://www.R-project.org). A value of p < 0.05 was considered as statistically significant.

## Results

### Discovery of up- or down-regulated plasma proteins in GBM patients by SWATH-MS analysis (Fig 1, steps 1–6)

**Fig 1:**
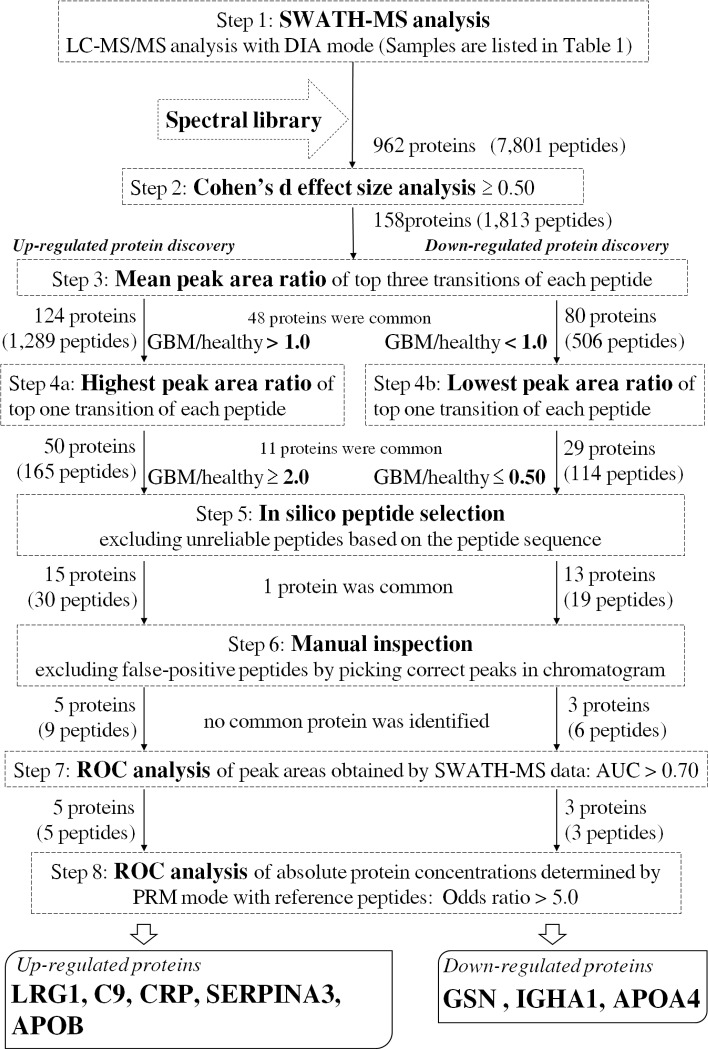
Flow diagram of GBM biomarker discovery.

Fig 1 illustrates the flow diagram of biomarker candidate selection among the identified proteins to diagnose GBM. Using the constructed spectral library, SWATH-MS analysis was performed for GBM plasma (n = 14) and healthy plasma (n = 15) (Table 1). SWATH data from a single injection for each GBM plasma and healthy plasma sample were analyzed, resulting in identification of 7,801 peptides (962 proteins) at peptide FDR < 1% with at least one sample (Fig 1, Step 1). In order to select up- or down-regulated proteins in GBM plasma, the top three transitions showing higher peak area were selected for each peptide. As clinically significant incidence is important for selection among the proteins identified, Cohen’s d effect size was adopted as an indicator of difference between two groups that is unaffected by sample size. We obtained 1,813 peptides (158 proteins) which gave an effect size equal to or greater than 0.50 (Fig 1, Step 2) in all top three transitions. The mean value of the peak area ratio between GBM and healthy plasma was obtained from the top three transitions. As a result, 1,289 peptides gave a mean peak area ratio of greater than 1.0 in all top three transitions, representing 124 up-regulated proteins (Fig 1, Step 3), and 506 peptides gave a mean peak area ratio of smaller than 1.0 in all top three transitions, representing 80 down-regulated proteins (Fig 1, Step 3). Among these proteins, 48 were identified as both up-regulated and down-regulated, judging on the basis of different peptide fragments from the same protein. Furthermore, the peak area ratio between GBM and healthy plasma was obtained from the top one transition with the highest peak area. As a result, 165 peptides showed a highest peak area ratio equal to or greater than 2.0, representing 50 up-regulated proteins (Fig 1, Step 4a), and 114 peptides showed a lowest peak area ratio equal to or smaller than 0.50, representing 29 down-regulated proteins (Fig 1, Step 4b). In order to exclude unreliable peptide candidates, in silico peptide selection criteria reported previously [30] were adopted for the sequences of all candidate peptides (Fig 1, Step 5). We selected 30 peptides (15 proteins) and 19 peptides (13 proteins) as up-regulated and down-regulated, respectively. One protein was both up- and down-regulated as judged from different peptide fragments. Then, manual inspection was performed for the LC-MS/MS chromatogram of the top three transitions in order to confirm correct peak detection. We excluded 10 up-regulated and 10 down-regulated proteins, as these proteins did not meet the selection criteria shown in Fig 1 after picking correct peaks. Finally, nine and six peptides were selected to represent five up-regulated and three down-regulated proteins, respectively (Fig 1, Step 6).

### ROC analysis of biomarker candidate proteins selected by SWATH-MS analysis in GBM plasma (Fig 1, step 7)

ROC analysis of eight candidate proteins selected at Step 6 (Fig 1) was performed for the peak area obtained by SWATH-MS analysis of healthy plasma (n = 15) and GBM plasma (n = 14). The values of area under the curve (AUC) are listed in Table 3, together with p value in Student’s t-test and Cohen’s d effect size. The AUC values of complement component C9 (C9), leucine-rich alpha-2-glycoprotein (LRG1), gelsolin (GSN) and Ig alpha-1 chain C region (IGHA1) were greater than 0.80, while the AUC values of all candidate proteins were greater than 0.70 (Table 3).

**Table 3:** Summary of the differentially expressed proteins identified in plasma analyzed by SWATH-MS analysis.

Protein	Peptide	AUC	p value	Effect size
**Up-regulated proteins**				
Complement component C9	LSPIYNLVPVK	0.871	7.35×10^−4^	1.59
	VVEESELAR*	0.867	2.33×10^−3^	1.40
Leucine-rich alpha-2-glycoprotein	ALGHLDLSGNR*	0.867	1.61×10^−3^	1.45
	DLLLPQPDLR	0.867	3.74×10^−3^	1.32
C-reactive protein	ESDTSYVSLK*	0.793	6.96×10^−2^	0.761
Apolipoprotein B-100	LATALSLSNK*	0.790	9.69×10^−3^	1.13
Alpha-1-antichymotrypsin	DSLEFR	0.771	2.40×10^−2^	0.977
	ITLLSALVETR	0.733	2.60×10^−2^	0.960
	NLAVSQVVHK*	0.729	3.28×10^−2^	0.910
**Down-regulated proteins**				
Gelsolin	TGAQELLR	0.962	3.16×10^−5^	2.03
	HVVPNEVVVQR*	0.876	2.37×10^−4^	1.57
Ig alpha-1 chain C region	DASGVTFTWTPSSGK*	0.862	8.39×10^−4^	1.40
Apolipoprotein A-IV	LTPYADEFK	0.814	2.82×10^−3^	1.22
	IDQNVEELK	0.800	2.13×10^−3^	1.32
	LEPYADQLR*	0.790	2.62×10^−3^	1.23

Receiver operating characteristics analysis and Student’s t-test were performed for the peak areas obtained by SWATH mass spectrometry (SWATH-MS) analysis of healthy plasma (n = 15) and glioblastoma plasma (n = 14). Peptides shown in bold with an asterisk (*) were selected as the most reliable peptide representing each protein for quantitative targeted absolute proteomics analysis based on the peak shape of chromatograms. AUC, area under the curve

### Accurate absolute quantification of biomarker candidate proteins for diagnosing GBM (Fig 1, step 8)

**Fig 2:**
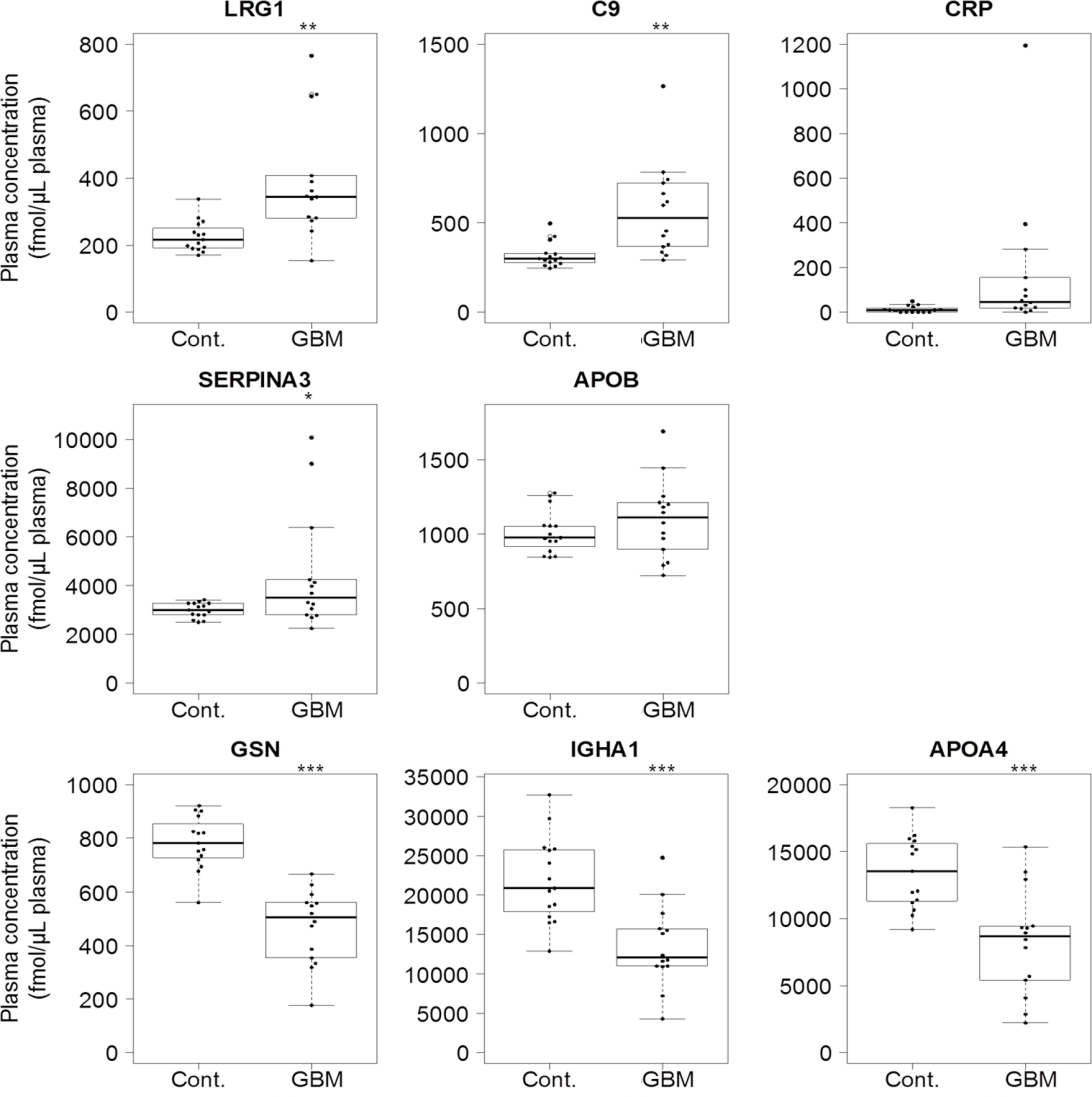
Box plot showing the plasma levels of each differentially expressed protein in GBM plasma (n = 14) compared with healthy plasma (n = 15). Each dot represents the protein level of an individual sample. In box plots, the band inside the box represents the median. The bottom and top of the box represent the first and third quartiles. The whiskers reflect the minimum and maximum values that fall within 1.5 times the interquartile range. Any data not included between the whiskers is an outlier. *, p < 0.05; **, p < 0.01; ***, p < 0.001. Cont, Healthy controls; GBM, glioblastoma.

As we cannot exclude the possibility of false-positive results caused by sample-dependent ion-suppression in MS/MS analysis, we confirmed the results shown in Table 3 by means of QTAP analysis. The GBM plasma (n = 14) and healthy plasma (n = 15) (Table 1) were analyzed by nanoLC-Triple TOF 5600 using the PRM mode in the presence of stable isotope-labelled peptides for the respective peptide identification; this enabled us to obtain the absolute protein concentration in plasma. The peptides shown in bold with an asterisk (*) in Table 3 were used for the QTAP analysis. The PRM transitions for QTAP analysis of each peptide are shown in S1 Table. The expression of candidate proteins listed in Table 3 was confirmed by the QTAP analysis (Table 4). The absolute protein concentrations in each plasma sample are shown in S2 and S3 Tables. Fig 2 illustrates box plots comparing the absolute concentration of biomarker candidate proteins between GBM patients and healthy controls. The protein concentrations of LRG1, C9 and alpha-1-antichymotrypsin (SERPINA3) in the GBM plasma were significantly greater than those in control plasma, while the protein concentrations of GSN, IGHA1, and apolipoprotein A-IV (APOA4) in the GBM plasma were significantly smaller than those in control plasma. ROC analysis of the eight candidate proteins selected at Step 7 (Fig 1) was performed for the absolute protein concentrations obtained by QTAP analysis of healthy plasma (n = 15) and GBM plasma (n = 14) (S2 Fig). Table 4 summarizes the AUC of ROC analysis with 95% confidence interval, threshold, sensitivity, specificity and odds ratio for all eight proteins selected at Step 7. The AUC values of LRG1, C9, CRP, GSN, IGHA1, and APOA4 were greater than 0.80 (Fig 1, Step 8). The odds ratio of each of the eight proteins was greater than 5.0. Accordingly, LRG1, C9, CRP, SERPINA3, and apolipoprotein B-100 (APOB) were selected as up-regulated biomarker candidate proteins and GSN, IGHA1 and APOA4 were selected as down-regulated biomarker candidate proteins for the diagnosis of GBM (Fig 1, Step 8).

**Table 4:** Summary of the differentially expressed proteins in plasma validated by QTAP analysis.

Protein	AUC (95%CI)	Threshold (fmol/μL plasma)	Sensitivity (%)	Specificity (%)	Odds ratio	p value	Effect size		Fold change in plasma			Cyst fluid		Fold change in tissue
**Up-regulated proteins**														
Leucine-rich alpha-2-glycoprotein	0.883 (0.735–1)	272	85.7	86.7	39.1	3.84×10^−3^		1.31	1.73	±	0.85		+	3.04
Complement component C9	0.886 (0.768–1)	335	85.7	80.0	24.0	3.80×10^−3^		1.32	1.78	±	0.91		+	5.18
C-reactive protein	0.833 (0.682–0.985)	13.0	85.7	73.3	16.5	8.49×10^−2^		0.718	13.9	±	31.0		+	0.299
Alpha-1-antichymotrypsin	0.695 (0.484–0.907)	3293	57.1	86.7	8.68	4.76×10^−2^		0.839	1.47	±	0.82		+	13.0
Apolipoprotein B-100	0.586 (0.359–0.813)	1068	57.1	80.0	5.32	2.86×10^−1^		0.416	1.09	±	0.30		+	2.46
**Down-regulated proteins**														
Gelsolin	0.986 (0.954–1)	672	100	93.3	∞	1.54×10^−7^		2.61	0.601	±	0.193		+	1.41
Ig alpha-1 chain C region	0.891 (0.769–1)	16119	78.6	93.3	51.1	2.32×10^−4^		1.58	0.617	±	0.282		+	4.22
Apolipoprotein A-IV	0.871 (0.737–1)	9859	78.6	93.3	51.1	2.51×10^−4^		1.57	0.612	±	0.316		+	14.3

Receiver operating characteristics (ROC) analysis and Student’s t-test were performed for the absolute protein concentrations obtained by quantitative targeted absolute proteomics (QTAP) analysis of healthy plasma (n = 15) and glioblastoma (GBM) plasma (n = 14). Thresholds were determined as the points with minimum distance from 100% sensitivity and 100% specificity in the ROC curve for GBM plasma (n = 14) and healthy plasma (n = 15). AUC is the area under the ROC curve, and the range of 95% confidence interval (CI) is shown. Odds ratio was calculated as %sensitivity × %specificity / (100−%sensitivity) × (100−%specificity). The fold changes of mean plasma concentrations between healthy controls (n = 15) and GBM patients (n = 14) were determined by QTAP analysis. In the cyst fluid column, + indicates any peptide(s) derived from the protein was identified in cyst fluid by data-dependent acquisition analysis. The fold changes of mean expression levels in cytosol fractions between GBM tissues (n = 14) and noncancerous brain tissues (n = 2) were determined by QTAP analysis.

### Quantification of biomarker candidate protein expression in GBM tissues

It is also important to examine the origin of biomarker candidate proteins selected by SWATH-MS and QTAP analysis (Table 4). Key questions are whether or not the up-regulated proteins are produced in GBM tissues in a greater amount than in noncancerous brain tissues, and whether or not they are secreted into cyst fluid. We performed QTAP analysis for the cytosol fractions of GBM tissues and noncancerous brain tissues. The expression levels of candidate proteins listed in Table 4 in cytosol fractions of GBM tissues and noncancerous brain tissues were determined. The ratios obtained (Table 4) indicate that all protein candidates, except for CRP, are more highly expressed in the cytosol fraction of GBM tissues than in that of noncancerous brain tissues. As shown in Table 4, all biomarker candidate proteins were detected in cyst fluid of GBM patients by DDA analysis. However, one peptide (GYSIFSYATK) derived from CRP, 243 peptides derived from APOB, and 29 peptides derived from APOA4 were identified in cyst fluid, instead of the peptides derived from CRP, APOB, and APOA4 shown in Table 3. A peptide derived from CRP was identified in IEF-fractionated cyst fluid, while peptides derived from all biomarker candidates, except for CRP, were identified in both unfractionated and IEF-fractionated cyst fluid.

### Correlation of plasma protein concentrations with total protein amounts in tumor tissues, tumor size, PFS, or OS among the GBM patients

**Fig 3:**
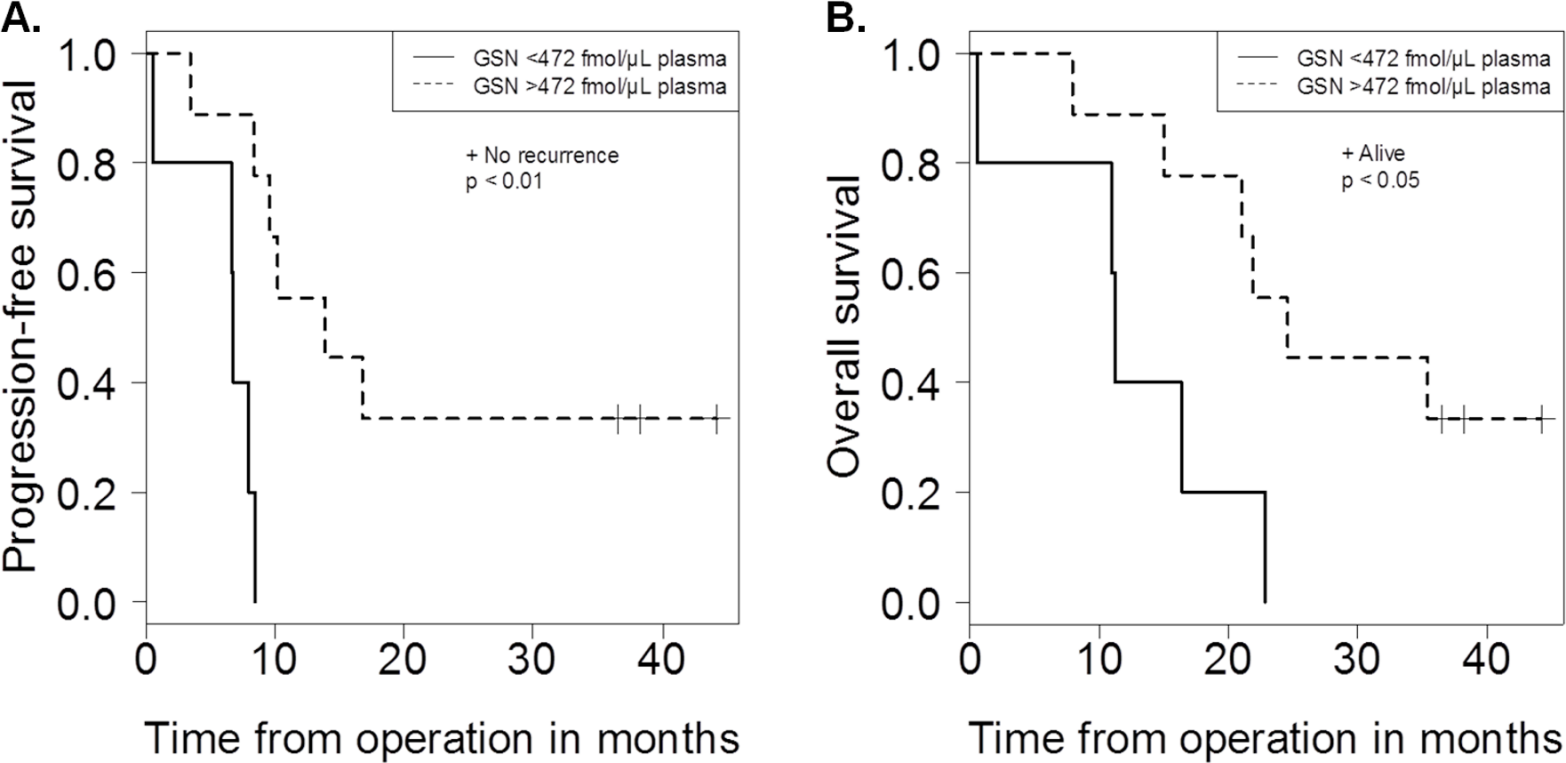
**Kaplan–Meier curve of progression-free survival time (PFS) (A) and overall survival time (OS) (B) in patients with glioblastoma (GBM) showed prognostic significance of gelsolin (GSN).** GBM patients were classified into two categories on the basis of GSN level: low (0–472 fmol/μL plasma) and high (> 472 fmol/μL plasma). Mean GSN plasma level in GBM patients was selected as the cut-off point. (A) PFS interval was determined as the interval between the date of initial operation and the date of patient’s recurrence or determined endpoint (for those no recurrent on August 1, 2015). (B) OS interval was determined as the interval between the date of the initial operation and date of patient’s death or determined end point (for those alive on August 1, 2015).

We also analyzed the correlation between concentrations of biomarker candidate proteins in plasma and total amounts of biomarker candidate proteins in cytosol fraction of GBM patients. The correlation coefficients are listed in Table 5 and the results for CRP, C9, LRG1, SERPINA3, and GSN are shown in S3 Fig. Pearson correlation analysis showed a significant positive correlation of plasma protein concentrations with total protein amounts in the tumor tissues for CRP (r = 0.974; p = 4.63×10^−9^, n = 14), C9 (r = 0.813; p = 4.07×10^−4^, n = 14), LRG1 (r = 0.802; p = 5.60×10^−4^, n = 14), and SERPINA3 (r = 0.597; p = 2.42×10^−2^, n = 14) (Table 5, S3 Fig). To investigate the relationships between biomarker candidates and GBM biology, correlations between the concentrations of biomarker candidates in plasma and clinical presentation (tumor size, PFS, or OS) in GBM patients were examined. Pearson correlation analysis showed significant positive correlations of plasma protein concentrations with tumor size for CRP (r = 0.704; p = 4.95×10^−3^, n = 14), C9 (r = 0.673; p = 8.36×10^−3^, n = 14), and LRG1 (r = 0.731; p = 2.97×10^−3^, n = 14) (Table 5, S3 Fig), and a significant positive correlation of plasma protein concentrations with OS for GSN (r = 0.573; p = 3.21×10^−2^, n = 14) (Table 5, S3 Fig). In addition, PFS and OS probabilities in GBM patients with low or high biomarker candidate plasma levels were examined (S4 Fig). Mean biomarker candidate plasma levels in GBM patients were selected as the cut-off points, except for CRP. CRP plasma level of 47.6 fmol/μL plasma, corresponding to a cut-off point of 5 mg/L selected by Strojnik *et al*. [5], was selected as the cut-off point. GSN levels below 472 fmol/μL plasma were significantly related to poor prognosis in GBM patients (Fig 3).

**Table 5:** Correlation of plasma protein concentrations with the total protein amounts in the tumor tissues, tumor size, PFS, or OS among the GBM patients.

Protein	Correlation coefficient (R^2^)			
	Total protein amount in tumor tissue	Tumor size	PFS	OS
**Up-regulated proteins**				
C-reactive protein	0.948***	0.495**	0.214	0.108
Complement component C9	0.661***	0.452**	0.0638	0.00775
Leucine-rich alpha-2-glycoprotein	0.643***	0.534**	0.0627	0.0197
Alpha-1-antichymotrypsin	0.357*	0.184	0.00879	0.0220
Apolipoprotein B-100	0.0323	0.225	0.134	0.0148
**Down-regulated proteins**				
Gelsolin	0.0166	0.00188	0.253	0.330*
Apolipoprotein A-IV	0.00450	0.0903	0.113	0.163
Ig alpha-1 chain C region	0.00187	0.0166	0.00999	0.00676

Pearson correlational analysis was used to compare plasma protein concentrations and the total protein amounts in the tumor tissues, tumor size, progression-free survival time (PFS), or overall survival time (OS) among the glioblastoma (GBM) patients. We multiplied cytosol protein concentrations by tumor size to calculate the total protein amounts in the tumor tissues.

*, p < 0.05

**, p < 0.01

***, p < 0.001.

## Discussion

By using the combination of SWATH-MS analysis and QTAP analysis, we identified eight biomarker candidates in plasma of GBM patients (Table 4). LRG1, C9, CRP, SERPINA3, and APOB were identified as up-regulated biomarker candidates, compared with the healthy plasma, while GSN, IGHA1, and APOA4 were identified as down-regulated biomarker candidates. This study is the first to quantify absolute plasma concentrations of LRG1, C9, SERPINA3, GSN, IGHA1, and APOA4 in GBM patients, and to show that their expression levels are significantly different (p < 0.05) from those in healthy controls.

It is also important to confirm the origin of up-regulated biomarker candidates. We performed QTAP analysis for cytosol fractions of GBM tissues and noncancerous brain tissues, and DDA analysis for cyst fluid. All the candidate proteins, except for CRP, were more highly expressed in cytosol of GBM tissues than in cytosol of noncancerous brain tissues (Table 4). SERPINA3 is known to be elevated in glioma tissues at both the mRNA and protein levels, compared with noncancerous brain tissues [31]. C9 is also known to be elevated in microsomal fraction of GBM tissues than in microsomal fraction of noncancerous brain tissues by isobaric tags for relative and absolute quantitation [32]. Immunostaining for CRP or monomeric CRP is often associated with neutrophils at inflammatory sites [33,34]. Generation of biologically active peptides from CRP after in vitro degradation by activated neutrophils or neutrophil proteases has been observed [35–37]. Expression of CRP in the cytosol of GBM tissues was lower than that in the cytosol of noncancerous brain tissues (Table 4), indicating the possibility that degraded CRP in GBM tissues is exported into circulation. Also, all the biomarker candidates were detected in cyst fluid (Table 4). Moreover, when we analyzed the correlations between the concentrations of biomarker candidates in plasma and the total amounts of biomarker candidates in cytosol of GBM patients, Pearson correlation analysis showed significant positive correlations for CRP, C9, LRG1, and SERPINA3 (Table 5, S3 Fig), indicating the possibility that the concentrations of the biomarker candidates in GBM plasma reflect the amounts in the GBM tissues. It is reported that the GBM-secreted inflammatory cytokine interleukin (IL)-6 acts on liver cells, inducing them to secrete high levels of CRP, which reaches the GBM tumor through the blood circulation and is accumulated in tumor tissues [38]. Taken together, the results indicate the possibility that C9, LRG1, and SERPINA3 in plasma are derived from the GBM tissues.

To investigate the relationships between biomarker candidates and GBM biology, we examined the correlations between the concentrations of biomarker candidates in the plasma and clinical presentation (tumor size, PFS, or OS) in GBM patients. Pearson correlation analysis showed a significant positive correlation between plasma protein concentrations and tumor size for CRP, C9, and LRG1 (Table 5, S3 Fig). Preoperative serum CRP levels are reported to be associated with tumor size in non-small cell lung cancer [39]. It is reported that CRP promotes endothelial cell survival by acting on microglial cells and promoting tumor angiogenesis [38]. There is recent evidence that LRG1 is induced by IL-6 and synergistically up-regulated with either IL-1β or tumor necrosis factor-α in a pattern similar to that exhibited by type 1 acute-phase proteins in human hepatoma HepG2 cells [40]. Previous study has also shown that up-regulation of LRG1 can promote endothelial cell proliferation and angiogenesis via the transforming growth factor-β signaling pathway [41]. Silencing the expression of LRG1 is reported to suppress the growth of GBM U251 cells in vitro and in vivo [42]. Taken together, a positive correlation between these protein levels and tumor size might be reasonable, because a large tumor cell burden is likely to increase inflammatory cytokine levels, stimulating these protein production.

In this study, we identified increased plasma levels of LRG1, APOB, and acute-phase proteins including C9, CRP, and SERPINA3 [33] in GBM patients. Acute-phase proteins are associated with various types of cancers, as well as other clinical conditions, and may be a result of inflammatory responses. Up-regulation of LRG1 is associated with multiple types of tumors [43–45]. CRP was reported to be elevated in blood of patients with GBM [38], various types of cancers [46,47] and other pathological conditions, for example autism [48]. C9 and SERPINA3 are elevated in blood of patients with various types of cancers [49,50]. C9 is known to be a terminal member of the cell killing process via a membrane-attack complex consisting of C5b, C6, C7, C8 and C9 [51]. C3, which is included in three pathways leading to the activation of membrane-attack complexes on the target cell, is produced by rat hepatoma cells in response to inflammatory cytokines, such as IL-1 and tumor necrosis factor [52]. SERPINA3 is synthesized primarily by hepatocytes, bronchial epithelial cells and monocytes but is also expressed in a variety of organs such as kidney, brain and prostate [53]. Inflammatory cytokines, like IL-6, stimulate the synthesis of SERPINA3 in human hepatocytes [54]. Moreover, SERPINA3 is overexpressed in invasive and metastatic melanomas, compared to normal nevi and melanoma-in-situ [55]. Knockdown of SERPINA3 declined melanoma migration and invasion abilities. Multiple steps are involved in invasion and metastasis of malignant cells to distant tissues, including cancer cell attachment to extracellular matrix, degradation of extracellular matrix components and subsequent infiltration into adjacent normal tissue [56], and therefore proteases such as matrix metalloproteinases are considered as key factors [57,58]. Moreover, protease inhibitors are generally considered to have an anti-malignant role [59]. However, some serine protease inhibitors appear to be regulated in various tumors, indicating a potential role in tumor progression [60]. APOB was reported to be elevated in blood of patients with some early-onset inflammatory diseases [61,62]. However, this mechanism is unclear. Although IL-6 induced a marked increase in APOB mRNA levels in HepG2 cells, it lowered the accumulation of APOB protein levels in the culture medium [63].

In this study, we identified decreased plasma protein levels of GSN, IGHA1, and APOA4 in GBM patients. GSN was reported to be decreased in cerebrospinal fluid of patients with astrocytomas [64] and in blood of patients with various types of cancers [65,66] and other pathological conditions, for example ischemic stroke [67]. GSN is a calcium-regulated actin-binding protein located in the cell cytoplasm or extracellular spaces [68]. Downregulation of extracellular GSN levels may result from the depletion of circulating GSN by scavenging of actin released from dying cells into the bloodstream [69,70]. Circulating actin presumably has toxic effects [71]. Our Pearson correlation analysis showed a significant positive correlation of plasma protein concentrations with OS for GSN (Table 5, S3 Fig). Moreover, GSN levels below 472 fmol/μL plasma were significantly related to poor prognosis in GBM patients by Kaplan–Meier analysis (Fig 3). GSN also affects cellular configuration, differentiation, motility, adhesiveness, and invasiveness, and regulates apoptosis in tissues [72]. Moreover, GSN overexpression is considered a poor prognostic factor in patients of oral cancer [73] and non-small cell lung cancer [74]. These reports have suggested that overexpression of GSN in tumors promotes the migratory capacity of tumor cells, thus enhancing their potential to invade both adjacent as well as distant tissues, resulting in a poor prognosis. Conversely, there is a report indicating that transfection of the GSN gene into human bladder cancer cells induced a significant decrease of tumorigenicity and colony-forming ability [75]. The function of GSN may depend on tumor type. Although the precise function of APOA4 in cancer is not known, APOA4 was reported to be decreased in blood of patients with various types of cancers [76,77]. APOA4 is primarily produced in intestinal enterocytes and secreted as chylomicrons and very-low-density lipoprotein apoprotein into the lymph [78].

In this work, the peptide derived from CRP (Table 3) was not identified in unfractionated plasma by DDA analysis, but SWATH-MS enabled us to identify CRP from an in-house spectral library generated from DDA data acquired in other experiments. Because the plasma flows through all organs, tissue-derived proteins become highly diluted during systemic circulation to a concentration range of ng/mL or below [79]. Based on the concentration range of candidate proteins used in this study (μg/mL) and that of currently used plasma cancer biomarkers (ng/mL) [80], it is evident that the concentration ranges of the two populations minimally overlap, suggesting that the proteomic strategies used in this study lacked the sensitivity to reliably detect potential biomarker proteins in lower concentration ranges.

In conclusion, we have identified eight biomarker candidates in plasma of the patients with GBM, IDH-wildtype by using the combination of SWATH-MS analysis and QTAP analysis. Although at least two peptides derived from five biomarker candidates (C9, LRG1, SERPINA3, GSN and APOA4) were shown to be differentially expressed in plasma by SWATH-MS analysis (Table 3), in order to make this study persuasive, it is desirable to quantify multiple peptides for each candidate protein using QTAP analysis to ensure complete digestion of the protein in the region of target peptides, and to verify the accuracy of the measurement. In addition, in order to confirm the correlations shown in this study (Table 5, S3 Fig), it will be necessary to conduct studies using plasma from larger number of GBM patients. Further investigations using plasma from patients with postoperative GBM, glioma, and other various types of cancer will be needed to establish the feasibility of clinical application of our findings.

## Supporting information

S1 Fig Venn diagram depicting overlaps of identified proteins by data-dependent acquisition. (TIF)

S2 Fig Receiver operating characteristics (ROC) curves of biomarker candidates for glioblastoma (GBM). (TIF)

S3 Fig Correlation of plasma protein concentrations with the total protein amounts in the tumor tissues (A), tumor size (B), overall survival time (OS) (C), or progression-free survival time (PFS) (D) among the glioblastoma (GBM) patients.
(TIF)

S4 Fig Kaplan–Meier curve of progression-free survival time (PFS) (A) and overall survival time (OS) (B) in patients with glioblastoma (GBM).
(TIF)

S1 Table Peptide probes and PRM transitions for QTAP analysis. (PDF)

S2 Table Plasma concentrations of up-regulated biomarker candidates in the GBM patients and healthy controls. (PDF)

S3 Table Plasma concentrations of down-regulated biomarker candidates in the GBM patients and healthy controls. (PDF)
