# Supplementary figures and images for: Identification of blood biomarkers in glioblastoma by SWATH mass spectrometry and quantitative targeted absolute proteomics
Source: PLoS One. 2018 Mar 7;13(3):e0193799. doi: 10.1371/journal.pone.0193799 (PMC5841790; doi:10.1371/journal.pone.0193799)

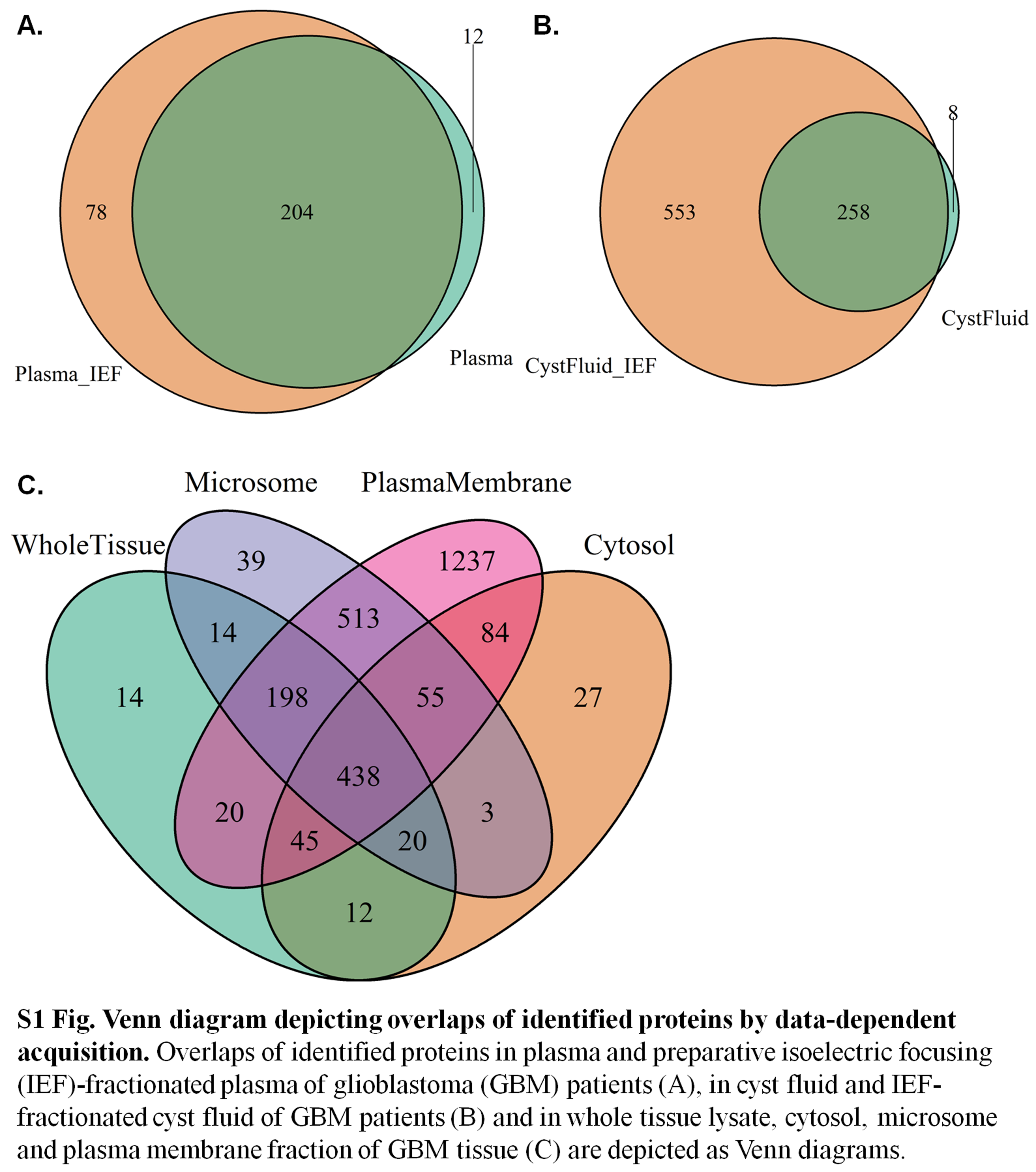

Supplement: S1 Fig — (TIF) [file pone.0193799.s001.tif]

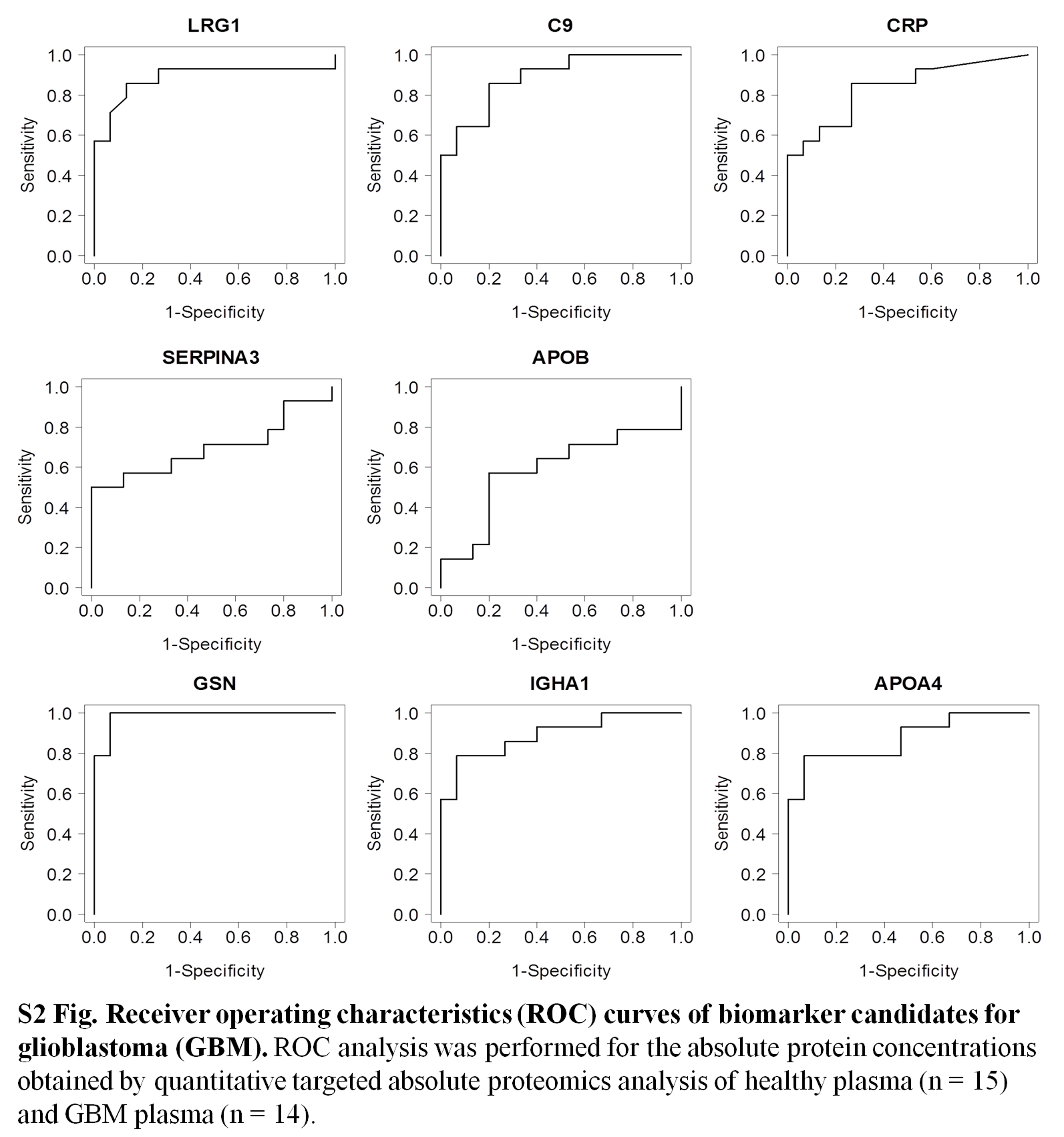

Supplement: S2 Fig — (TIF) [file pone.0193799.s002.tif]

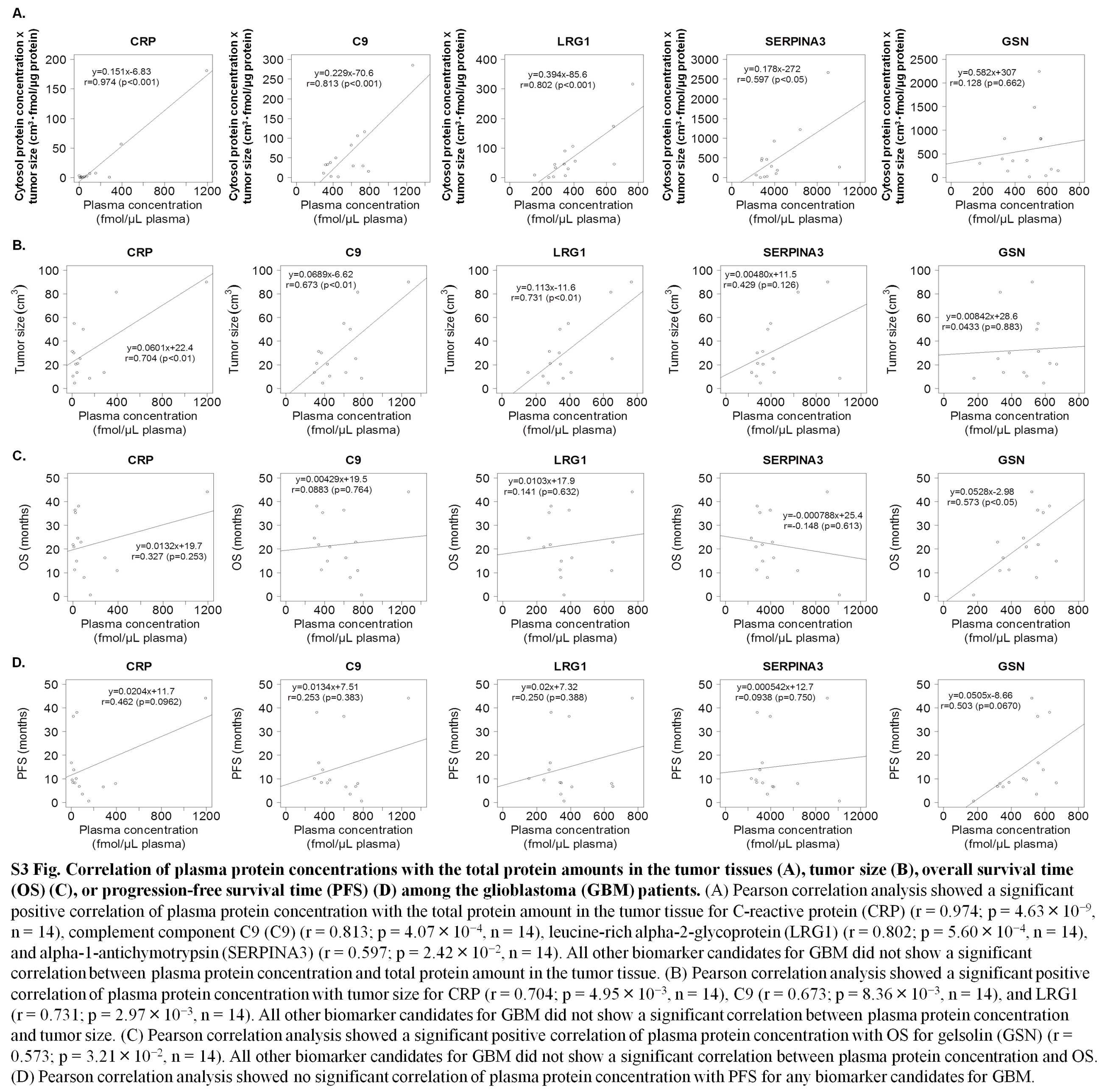

Supplement: S3 Fig — Correlation of plasma protein concentrations with the total protein amounts in the tumor tissues (A), tumor size (B), overall survival time (OS) (C), or progression-free survival time (PFS) (D) among the glioblastoma (GBM) patients. (TIF) [file pone.0193799.s003.tif]

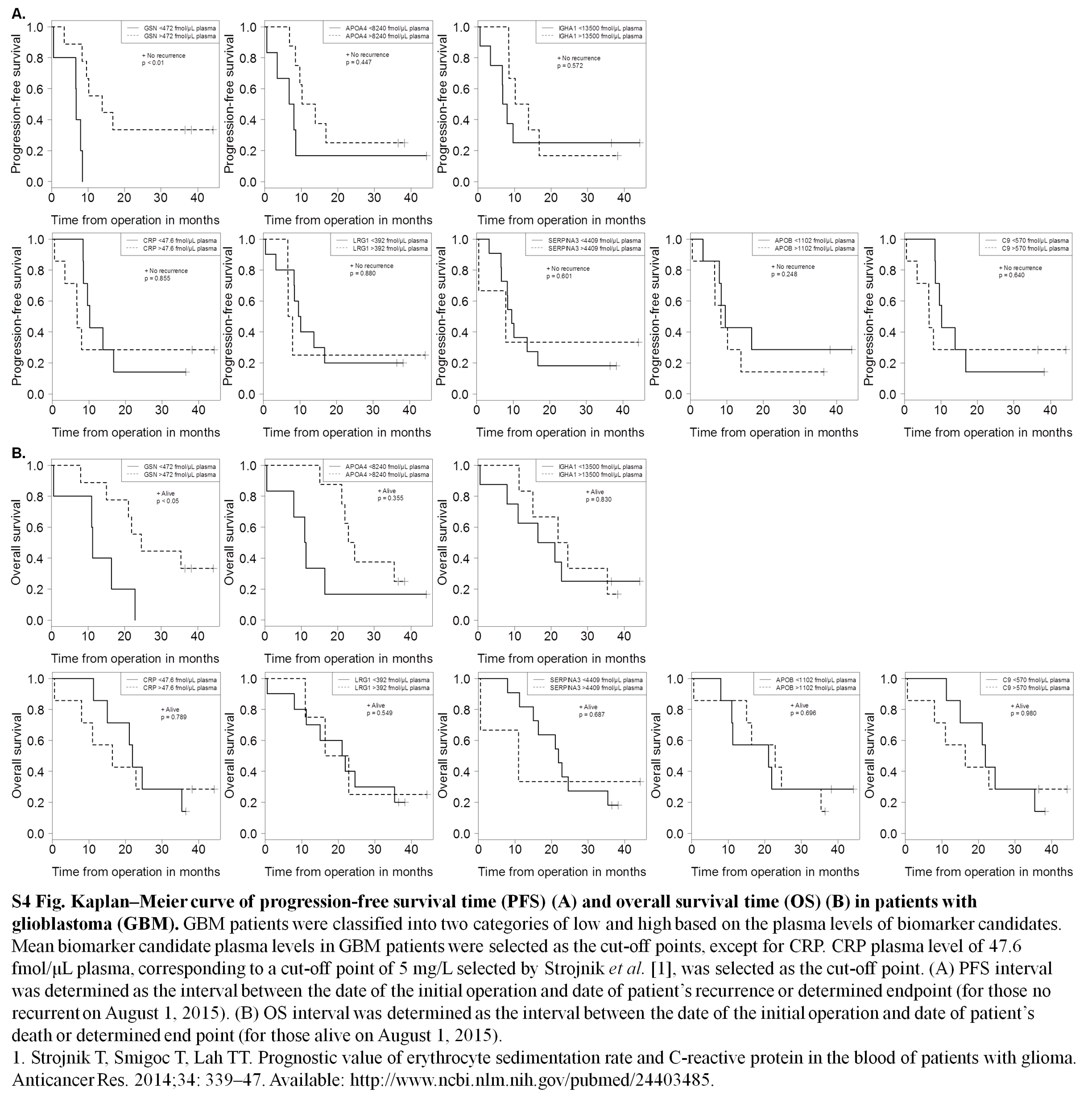

Supplement: S4 Fig — Kaplan–Meier curve of progression-free survival time (PFS) (A) and overall survival time (OS) (B) in patients with glioblastoma (GBM). (TIF) [file pone.0193799.s004.tif]
