# Supplementary material for: Identification of blood biomarkers in glioblastoma by SWATH mass spectrometry and quantitative targeted absolute proteomics
Source: PLoS One. 2018 Mar 7;13(3):e0193799. doi: 10.1371/journal.pone.0193799 (PMC5841790; doi:10.1371/journal.pone.0193799)
Supplement: S1 Table — (PDF) [file pone.0193799.s005.pdf]

S1 Table. Peptide probes and PRM transitions for QTAP analysis.

Uni-Prot Accession No.	Protein	Gene	Probe Sequence	PRM transition				
				Q1 (m/z)	TOF range (m/z±0.025)			
					1	2	3	4
P01011	Alpha-1-antichymotrypsin	SERPINA3	NLAVSQVVHK	365.5	697.399	200.140	284.172	383.240
			NLAVSQVVH**K***	368.2	705.413	200.140	292.186	391.254
P06727	Apolipoprotein A-IV	APOA4	LEPYADQLR	552.8	862.442	431.725	602.326	765.389
			LEPYADQL**R***	557.8	872.450	436.729	612.334	775.397
P04114	Apolipoprotein B-100	APOB	LATALSLSNK	509.3	661.388	548.304	185.129	286.176
			LATALSLSN**K***	513.3	669.402	556.318	185.129	286.176
P02748	Complement component C9	C9	VVEESELAR	516.3	704.357	171.149	575.315	199.144
			VVEESELA**R***	521.3	714.366	171.149	585.323	199.144
P02741	C-reactive protein	CRP	ESDTSYVSLK	564.8	696.393	347.229	609.361	446.297
			ESDTSYVSL**K***	568.8	704.407	355.243	617.375	454.312
P06396	Gelsolin	GSN	HVVPNEVVVQR	425.9	501.314	676.342	402.246	336.203
			HVVPNEVVVQ**R***	429.2	511.323	676.342	412.254	336.203
P01876	Ig alpha-1 chain C region	IGHA1	DASGVTFTWTPSSGK	770.9	475.251	576.299	430.193	331.125
			DASGVTFTWTPSSG**K***	774.9	483.265	584.313	430.193	331.125
P02750	Leucine-rich alpha-2-glycoprotein	LRG1	ALGHLDLSGNR	384.9	433.215	661.326	346.183	607.320
			ALGHLDLSGN**R***	388.2	443.224	671.335	356.192	607.320

Bold letters with asterisks indicate amino acid residues labeled with stable isotope (^{13}C and ^{15}N). Four parallel reaction-monitoring (PRM) transitions (Q1/TOF-1, Q1/TOF-2, Q1/TOF-3, and Q1/TOF-4), each of which consisted of the m/z value of precursor ion (Q1) and the m/z value (TOF1-4) of the product ion with a range of m/z±0.025, were set for each peptide. Collision energy (CE) for each targeted peptide was calculated from Eq. 1. In quantitative targeted absolute proteomics

(QTAP) analysis, CE of stable isotope-labeled peptides was calculated from Eq. 1 using precursor ion m/z of paired standard peptides. Collision energy spread was set at 5 eV.

$$\mathrm{CE} = 0.044 \times (\mathrm{precursor\ ion\ m/z}) + 4 \quad \text{(Eq. 1)}$$

The target peptide to be quantified were selected by the m/z value of Q1 using the MRM-High Resolution mode of Triple TOF 5600 (SCIEX, Framingham, Massachusetts). An auto analysis system established in our laboratory was used to extract the data set of each target peptide with the four transitions in the MRM-High Resolution analysis and to determine the quantitative value of the target peptide.
