# Supplementary material for: Identification of blood biomarkers in glioblastoma by SWATH mass spectrometry and quantitative targeted absolute proteomics
Source: PLoS One. 2018 Mar 7;13(3):e0193799. doi: 10.1371/journal.pone.0193799 (PMC5841790; doi:10.1371/journal.pone.0193799)
Supplement: S2 Table — (PDF) [file pone.0193799.s006.pdf]

S2 Table. Plasma concentrations of up-regulated biomarker candidates in the GBM patients and healthy controls.

Subject	Plasma concentration (fmol/µL plasma, mean ± SEM)				
	LRG1	C9	CRP	SERPINA3	APOB
P1	408 ± 28	619 ± 35	283 ± 21	4256 ± 113	1199 ± 29
P2	272 ± 9	378 ± 25	21.4 ± 5.4	3058 ± 137	1146 ± 53
P3	362 ± 12	785 ± 41	154 ± 8	10086 ± 49	1184 ± 17
P4	765 ± 39	1267 ± 37	1193 ± 24	9017 ± 26	724 ± 19
P5	337 ± 23	368 ± 8	13.7 ± 1.8	2775 ± 99	809 ± 32
P6	644 ± 27	741 ± 53	393 ± 21	6395 ± 24	901 ± 37
P7	651 ± 10	724 ± 38	72.0 ± 6.7	4147 ± 117	1693 ± 42
P8	242 ± 8	454 ± 23	6.82 *	2692 ± 67	1078 ± 27
P9	285 ± 17	318 ± 17	51.5 ± 3.0	2812 ± 55	975 ± 36
P10	343 ± 12	663 ± 33	100 ± 10	3693 ± 98	1011 ± 33
P11	280 ± 10	338 ± 23	ULQ (<15.6)	3261 ± 78	790 ± 13
P12	391 ± 15	598 ± 40	16.8 ± 3.9	3965 ± 191	1212 ± 24
P13	346 ± 17	430 ± 35	32.5 ± 5.6	3305 ± 81	1448 ± 40
P14	154 ± 13	293 ± 12	41.5 ± 5.5	2264 ± 64	1256 ± 53
C1	207 ± 14	274 ± 21	6.25 ± 2.29	3370 ± 173	957 ± 8
C2	194 ± 7	325 ± 23	9.86 *	2837 ± 107	972 ± 28
C3	189 ± 4	279 ± 14	ULQ (<14.8)	2529 ± 60	954 ± 38
C4	198 ± 17	298 ± 22	11.6 ± 3.3	2947 ± 54	1058 ± 24
C5	191 ± 6	331 ± 13	9.14 ± 5.25	2995 ± 87	852 ± 39
C6	338 ± 27	304 ± 12	48.3 ± 4.3	3136 ± 99	1258 ± 45
C7	263 ± 7	311 ± 14	ULQ (<13.0)	2806 ± 52	1279 ± 44
C8	271 ± 16	407 ± 33	30.7 ± 6.0	3280 ± 100	976 ± 10
C9	169 ± 7	262 ± 9	ULQ (<13.2)	2588 ± 90	849 ± 13
C10	179 ± 9	292 ± 23	ULQ (<14.5)	3174 ± 129	1002 ± 18
C11	217 ± 13	259 ± 19	ULQ (<17.0)	2816 ± 81	844 ± 20
C12	239 ± 9	291 ± 13	10.5 ± 4.8	3426 ± 22	1053 ± 28
C13	230 ± 12	248 ± 18	ULQ (<13.1)	2489 ± 71	1053 ± 31
C14	280 ± 16	498 ± 15	34.0 ± 2.4	3270 ± 108	885 ± 22
C15	233 ± 7	425 ± 31	23.1 ± 6.1	3270 ± 110	1221 ± 24

Each value represents the mean±SEM (n=3-4 transitions) of the quantitative values determined by using 3 or 4 parallel reaction-monitoring transitions in one analysis. Asterisk represents the mean of the quantitative values obtained from two transitions. GBM, glioblastoma; SEM, standard error of mean
