# Supplementary material for: Identification of blood biomarkers in glioblastoma by SWATH mass spectrometry and quantitative targeted absolute proteomics
Source: PLoS One. 2018 Mar 7;13(3):e0193799. doi: 10.1371/journal.pone.0193799 (PMC5841790; doi:10.1371/journal.pone.0193799)
Supplement: S3 Table — (PDF) [file pone.0193799.s007.pdf]

S3 Table. Plasma concentrations of down-regulated biomarker candidates in the GBM patients and healthy controls.

Subject	Plasma concentration (fmol/µL plasma, mean ± SEM)		
	GSN	IGHA1	APOA4
P1	353 ± 9	11628 ± 686	2896 ± 191
P2	590 ± 14	15535 ± 1168	8448 ± 670
P3	178 ± 6	7183 ± 589	4083 ± 236
P4	521 ± 9	12338 ± 716	5712 ± 190
P5	386 ± 14	17675 ± 1137	5433 ± 461
P6	333 ± 4	10974 ± 390	2220 ± 162
P7	319 ± 10	4304 ± 240	9429 ± 681
P8	489 ± 19	11040 ± 87	9300 ± 458
P9	626 ± 10	15700 ± 784	15347 ± 1024
P10	549 ± 15	11804 ± 370	7827 ± 573
P11	560 ± 22	20119 ± 1297	12921 ± 847
P12	558 ± 14	10880 ± 627	9341 ± 587
P13	667 ± 22	24706 ± 2041	13474 ± 562
P14	473 ± 7	15109 ± 1161	8930 ± 403
C1	907 ± 11	17229 ± 1442	11985 ± 447
C2	783 ± 13	12869 ± 788	13539 ± 452
C3	884 ± 21	32685 ± 2247	15790 ± 1046
C4	754 ± 9	18577 ± 210	10268 ± 786
C5	922 ± 13	16673 ± 1172	16213 ± 1021
C6	901 ± 24	16528 ± 1342	15145 ± 733
C7	820 ± 7	22095 ± 1134	14878 ± 635
C8	694 ± 29	29756 ± 1877	12058 ± 934
C9	678 ± 23	20864 ± 1385	15432 ± 1219
C10	818 ± 21	25649 ± 2389	11224 ± 850
C11	562 ± 15	25856 ± 1327	10642 ± 662
C12	736 ± 20	18784 ± 711	11444 ± 351
C13	757 ± 14	26019 ± 1174	15990 ± 992
C14	826 ± 17	20537 ± 1245	18281 ± 914
C15	720 ± 16	24089 ± 1417	9198 ± 562

Each value represents the mean±SEM (n=3-4 transitions) of the quantitative values determined by using 3 or 4 parallel reaction-monitoring transitions in one analysis. GBM, glioblastoma; SEM, standard error of mean
